# Composting of rice straw with effective microorganisms (EM) and its influence on compost quality

**DOI:** 10.1186/1735-2746-10-17

**Published:** 2013-02-07

**Authors:** Mohd Lokman Che Jusoh, Latifah Abd Manaf, Puziah Abdul Latiff

**Affiliations:** 1 Department of Environmental Science, Faculty of Environmental Studies, Universiti Putra Malaysia, Selangor Darul Ehsan, Malaysia

**Keywords:** Effective microorganisms (EM), Rice straw, Composting, Nutrients contents, Heavy metals contents

## Abstract

This study aims to assess the effect of EM application on the composting process of rice straw with goat manure and green waste and to evaluate the quality of both compost treatments. There are two treatment piles in this study, in which one pile was applied with EM and another pile without EM. Each treatment was replicated three times with 90 days of composting duration. The parameters for the temperature, pH, TOC and C/N ratio, show that decomposition of organic matter occurs during the 90-day period. The *t*-test conducted shows that there is a significant difference between compost with EM and compost without EM. The application of EM in compost increases the macro and micronutrient content. The following parameters support this conclusion: compost applied with EM has more N, P and K content (P < 0.05) compared to compost without EM. Although the Fe in compost with EM is much higher (P < 0.05) than in the compost without EM, for Zn and Cu, there is no significant difference between treatments. This study suggests that the application of EM is suitable to increase the mineralization in the composting process. The final resultant compost indicated that it was in the range of the matured level and can be used without any restriction.

## Introduction

Rice (*Oryza sativa*) is the most important staple food for a large part of the world’s human population, especially in East and South Asia, Latin America, the Middle East, and the West Indies. It is the grain with the second-highest worldwide production, after maize (corn) [1]. In Malaysia, it is the main source of carbohydrate and covers about 10% of total plantation area. Besides producing rice seed, it also produces a large amount of waste by-product of which one is rice straw residue. It is estimated that from 700,000 hectares of rice plantation area in Malaysia, two million metric tonnes of rice straw will be produced in every planting season [2]. A major portion of this agricultural waste is disposed of by burning or is mulched in the rice fields. These wastes, if not properly handled, will cause many problems to farmers as well as to the environment. If rice straw is left in the field without proper management, it can cause the spreading of disease, which originates from the rice straw, such as stem disease and can also encourage the breeding of pests, especially rats. Burning is not the best way to deal with such waste, as it is harmful to the environment.

The abundance of rice straw as an organic waste can be converted to fertilizer throughout the process of composting. According to Tiquia and Tam [3], composting is a biological treatment that is cost-effective to treat different types of organic waste. Composting was the first concept for using effective microorganisms (EM) in environmental management. Crop residues and animal wastes have been effectively composted to produce biofertilizers. EM was developed by Professor Dr Teruo Higa in the 1970s at the Ryukyus University, Okinawa, Japan. Basically, this microbial solution was developed for natural or organic farming systems, however, with further research its uses have been expanded to resolve some environmental issues, through which it facilitates the reuse of most waste [4]. Research in Holland [5] and in Costa Rica [6] has highlighted that there is potential for compost from the waste of animals or crops from which the results are increasing yields of crops supplied with this material compared to the output of traditional organic systems. Composting of rice straw using effective microorganisms as an accelerator to speed up the composting process and increased nutrients in the compost has not been well documented. Therefore, it is essential to study the effect on the physical and chemical parameters during the process.

The aim of this study is to assess the effect of EM application on the composting process of rice straw with goat manure and green waste and also to evaluate the nutrient and heavy metals at the end of composting. This study is based on the hypothesis that the application of EM on rice straw will increase the microbial activity and thus, increase the composting rate. It also increases the mineralization process of compost through the enhanced microbial colonization and activity. To test these hypotheses, compost from rice straw residue was produced with applications of EM and compared to non-EM treatment.

## Materials and methods

### Composting process and sampling

In this study, rice straw is used as the main material for composting together with goat manure and green waste (vegetable and fruit waste from the market). The shredded rice straw was soaked in the water for 24 hours before the composting process. All of the raw materials were analysed for physiochemical, nutrients and heavy metals. Composting was carried out in a shaded area at the premises of Premium Agro Products Sdn. Bhd. In this experiment, there are two treatments—compost piles with EM (C_1_) and compost piles without EM (C_2_) for control. The mixtures used for all piles were arranged with the ratio as follows: 50% rice straw + 30% goat manure + 20% green waste, in which10 kg of rice straw was mixed with 6 kg of goat manure and 4 kg of green waste, and each treatment was replicated three times. In this study we used commercial EM (EM•1®), which contains lactic acid bacteria, yeast and phototrophic bacteria. The EM solution is required for the production of EMAS. EMAS is actually an activated EM suspension in a mixture of molasses (sugar cane) and non-chlorinated water or rice rinse water (which provides the minerals for the multiplication of the microorganisms). For the activation of EM · 1®, one part EM · 1® microbial inoculants and one part of molasses were mixed with 20 parts of chlorine-free water. This solution was then stored for three to five days in an air tight expandable container for fermentation. Built up gas was released once daily.

For piles with inoculation with EM, a 5% EM solution was applied to constitute 20% of the amount of water to be added to a given mixture. Water was then added until the moisture content reached 60% (wet basis) in each composting mixture. To retain the moisture and prevent excessive loss of heat, the heaps of composting material were then covered using plastic sheets. The moisture content was maintained at 50–60% by the addition of water throughout the active composting period by frequent checking. The mixtures were turned at 3-day intervals to maintain porosity and were composted for 90 days. The temperature was measured daily with a digital thermometer at random depths.

Compost samples were taken from each treatment at 0, 15, 30, 45, 60 and 90 days of the composting and were analysed for changes in the physical and chemical properties throughout the composting process. The samples taken from the piles were divided into two portions. One portion was dried to constant weight (60°C for 2 days) for chemical analysis. The dried samples were ground in mortar to pass through a 2 mm sieve and were stored in screw capped jars. The other portion was analysed for pH and total carbon.

### Physiochemical analysis

Moisture content was determined using the gravimetric method. Five grams of compost was dried in an oven for 24 h at 105°C (weighed and re-weighed until a constant weight was reached). The samples were then allowed to cool at room temperature before the final weight was taken. The preparation for pH was conducted according to Sundberg *et al.*[7] and the pH was determined using a digital electrode pH meter. The total carbon was determined by loss of weight using the ignition method and the total nitrogen was determined using the Kjeldahl method [8]. The Total Organic Carbon (TOC) was determined by wet digestion in K_2_Cr_2_O_7_ with concentrated H_2_SO_4_, digested on a 150°C preheated block for 30 minutes, and titrated for excess Cr_2_O^2−^_7_ with ferrous ammonium sulphate after cooling. The contents of the mineral elements for Zn, Cu and Fe were determined using the ISO11466 [9] standard method (aqua regia digestion method) and analysed by Atomic absorption spectroscopy (AAS). Determination of P and K was conducted using the In-House Method at ESPEK Research and Advisory Services.

### Statistical analysis

Statistical analysis was carried out by using Statistical Package for the Social Sciences (SPSS) 17.0. All results were tested for normal distribution and then analysed by using the paired-sample *t*-test.

## Results

To promote the growth of microbial populations as a way to increase the decomposition of organic matter during composting, a favourable environment or condition must be provided. The favourable conditions and appropriate loading of microbial nutrients, as well as the non existence of harmful toxic compounds, are essential to reach high microbial degradation. The physical and chemical characteristics of composting raw materials are presented in Table 1. In general, most of the values for the parameters for rice straw are within the ranges obtained by Iranzo *et al.*[10] except for the Cu for which the values are a little high. The pH values of rice straw (7.6) were also in a similar range as obtained by previous studies[11,12], as were the pH values for goat manure (7.1) and green waste (8.4), which indicates that these residues are non-acidic.

**Table 1:** Chemical and physical properties of raw feedstock (dry weight basin)

**Parameter**	**Rice straw**	**Goat manure**	**Green waste**
pH	7.60 ± 0.08	7.10 ± 0.08	6.50 ± 0.48
TOC (%)	39.20 ± 0.95	35.60 ± 1.20	15.30 ± 0.91
C/N ratio	61.30 ± 2.61	13.00 ± 1.11	8.40 ± 0.21
MC (%)	11.43 ± 0.77	58.00 ± 0.84	79.00 ± 1.35
N (%)	0.64 ± 0.07	2.55 ± 0.28	2.90 ± 0.35
P (%)	0.21 ± 0.02	0.24 ± 0.03	0.18 ± 0.04
K (%)	1.12 ± 0.11	1.81 ± 0.15	0.95 ± 0.06
Zn (mg kg^−1^)	38.40 ± 2.20	65.20 ± 2.03	64.30 ± 0.51
Cu (mg kg^−1^)	9.30 ± 0.67	19.30 ± 0.67	1.32 ± 0.26
Fe (mg kg^−1^)	129.20 ± 1.90	1003.00 ± 12.49	145.20 ± 1.70

*TOC*: Total Organic Carbon, *C/N ratio*: Carbon/Nitrogen ratio, *MC*: Moisture Content, *N*: Nitrogen, *P*: Phosphorous, *K*: Potassium, *Zn*: Zinc, *Cu*: Copper, and *Fe*: Iron.

**Figure 1:**
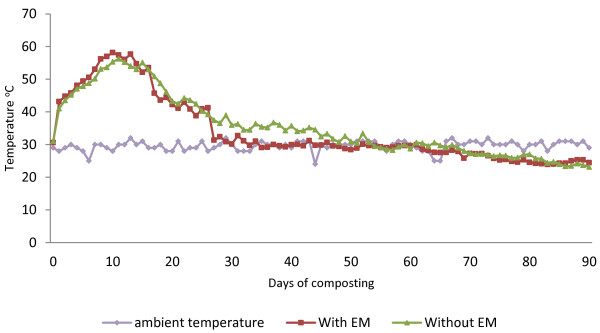
Changes in temperature during composting process.

The temperature profiles of two different composting treatments are shown in Figure 1. All treatments show an increase in temperature right after composting started. On Day 1, the temperature rose to 43.1°C from 30.6°C for treatment with EM (C_1_) and 41.0°C from 31.0°C for treatment without EM (C_2_). The piles treated with EM reached the highest peak values of 58.2°C on Day 10 compared to composting treatment without EM, for which it was 56.2°C on Day 11. This shows that all the composting treatments reached thermophilic temperature (>40°C). The thermophilic phase lasted for 23 days for treatment C_1_, whereas treatment C_2_ lasted for 30 days. Figure 1 shows that the temperature gradually decreased afterwards and finally stabilized near the ambient temperature at 30–35 days for composting treatment C_1_and 48–52 days for composting treatment C_2_.

**Figure 2:**
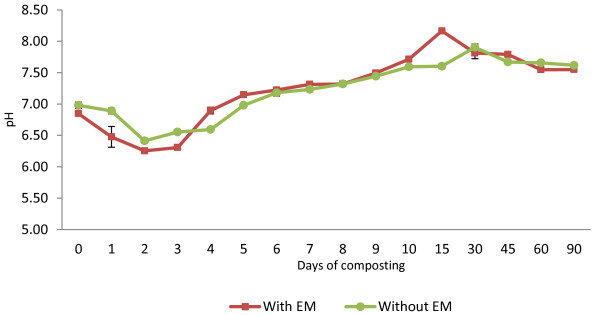
Changes in pH of composting mixtures with days.

The minimum pH recorded was around 6.25 for C_1_ and 6.41 for C_2_ on Day 2 decreasing from the initial values from 6.85 for C_1_ and 6.98 for C_2_. According to Figure 2, the initial pH values for both treatments tended to decrease in the first week and gradually increased and reached the highest peak, which was 8.16 on day 15 for compost C_1_ and 7.90 on day 30 for compost C_2_. Following that, the pH value declined to stabilize with the end result at 90 days being 7.55 for composting treatment C_1_ and 7.62 for composting treatment C_2_. This indicates a good quality compost and within the suggested range of 6–8.5, as has been reported by several studies [13].

**Figure 3:**
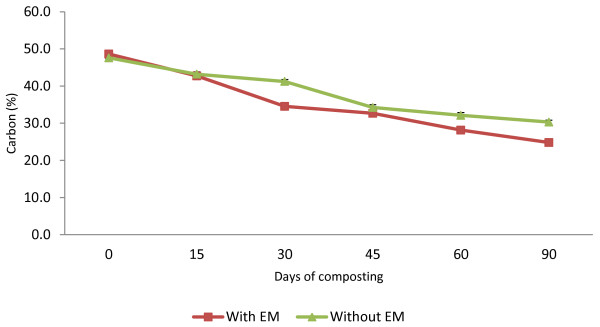
Changes in TOC of composting mixtures with days.

The TOC concentration (Figure 3) declined slightly for both treatments. The initial values of TOC were 48.6% for C_1_ and 47.6% for C_2_. Between the initial day of the composting period until Day 15 the decreasing values of TOC in both treatments were quite similar to each other, and, afterwards, C_1_ continued to decrease sharply until Day 30, while in C_2,_ the decrease in rates were slower. The decreasing percentage for C_1_ was 49.1% and 36.3% for C_2_.

**Figure 4:**
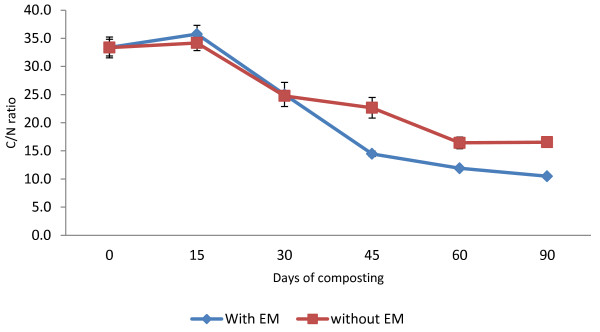
Changes in C/N ratio of composting mixtures with days.

One of the often used parameters to assess the rate of decomposition in the composting process is the C/N ratio, since it can reflect the maturity of the compost. Figure 4 shows the decrease in C/N values in both treatments due to the mineralization of organic matter. The initial C/N ratio for C_1_ was 32.4, while for C_2_ it was 34.0, and the final values of total C/N ratio after the 90-day composting period were 10.3 for C_1_ and 16.1 for C_2_. These results compare favourably with those of other research, such as Makan *et al.*[14], Roca-Peréz *et al.*[15] and Tumuhairwe *et al.*[16]. A C/N ratio of less than 20 is considered as mature and can be used without any restriction. Although a C/N ratio of 10 to 20 normally indicates as being in the range of the mature level, the higher ratio cannot be concluded, as the compost is not mature enough. This is because sometimes C is not in an available form. The C/N ratio in C_1_ compost drops 68% compared to C_2_, which is 53%. The *t*-test that was conducted indicates that there is a significant difference (p < 0.05) between compost treatments.

**Figure 5:**
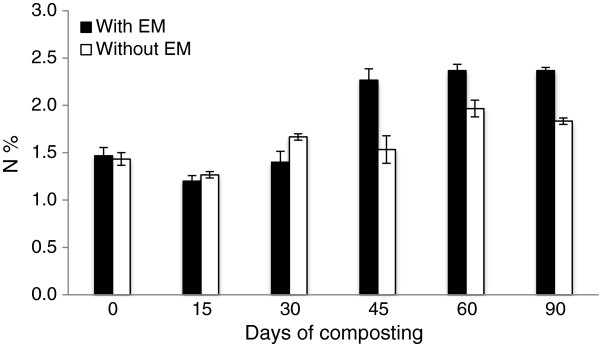
Changes in N content in composting mixtures with days.

**Figure 6:**
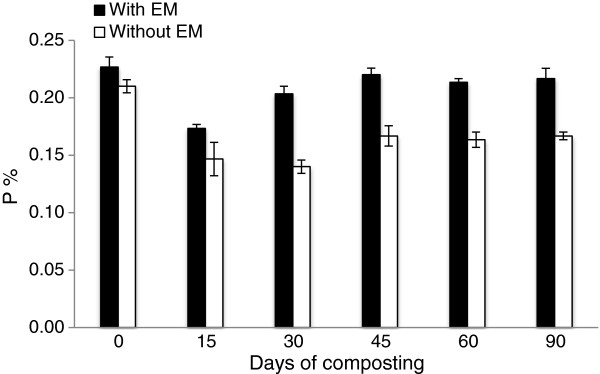
Changes in P content in composting mixtures with days.

**Figure 7:**
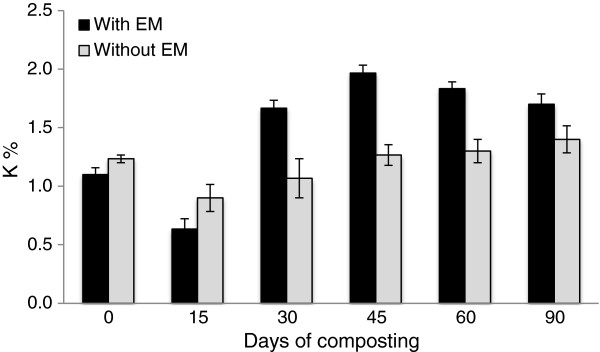
Changes in K content in composting mixtures with days.

The nutrient content for both of the treatments showed that N and K experienced an increase in the percentage at the end of composting while P showed a decreasing trend. The initial value of N in early composting was 1.5% for C_1_ and 1.4% for C_2_. At the end of composting both treatments showed an increase in total N content where C_1_ was 2.4% and C_2_ was 1.8% (Figure 5). The initial P value was 0.23% for C_1_ and 0.21% for C_2_. It was observed that the P content in the compost declined at the end of the composting period for both treatments. At the end of the composting the values for C_1_was 0.22% and 0.17% for C_2_ (Figure 6). The initial value of the K percentage on the initial day was 1.1% for C_1_ and 1.2% for C_2_. Between these two treatments, C_1_ achieved the highest values of K on day 45, which was 2.0%. At the end of the composting period, the K value in C_1_ was 1.7% and 1.4% for C_2_ (Figure 7).

**Figure 8:**
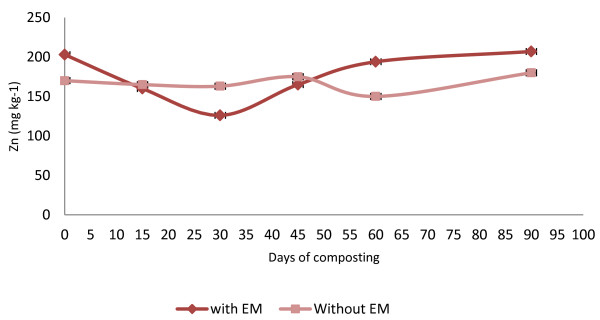
Changes in Zn of the composting mixtures during composting period.

**Figure 9:**
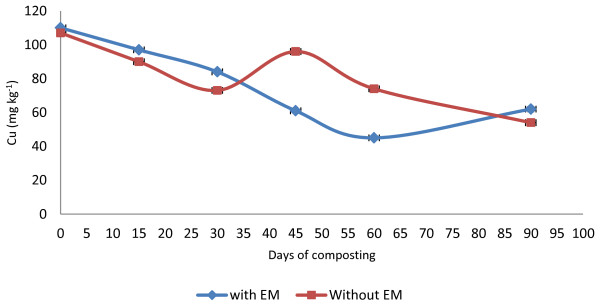
Changes in Cu of the composting mixtures during composting period.

**Figure 10:**
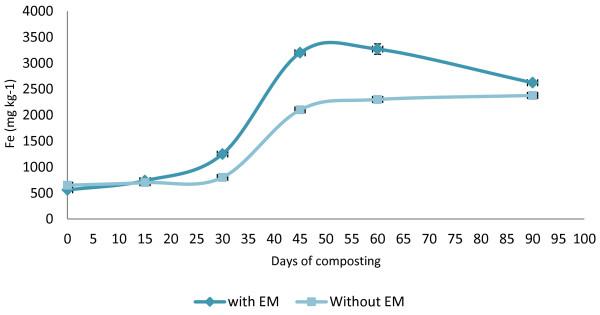
Changes in Fe of the composting mixtures during composting period.

The heavy metal (micronutrients) content in both of the treatments showed an increase in the amount except for the Cu, which decreased. The initial value for Zn was 203 mg kg^−1^ for C_1_ and 170 mg kg^−1^ for C_2_ (Figure 8). The final Zn value for C_1_was 207 mg kg^−1^ and 180 mg kg^−1^ for C_2_. For the Cu trend in compost C_1_, the Cu values (110 mg kg^−^1) decreased from the early days of composting to the end of the composting period (62 mg kg^−1^) (Figure 9). Whereas for the C_2_, the initial value was 107 mg kg^−1^ and decreased to 54 mg kg^−1^ at the end of composting. The increasing trend in Fe is shown in the initial value of 560 mg kg^−1^ in C_1_ and 650 mg kg^−1^ for C_2_ with the result of 2624 mg kg^−1^ for C_1_ and 2379 mg kg^−1^ for C_2_ (Figure 10).

## Discussion

The increase in temperature during the composting process was caused by the heat generated from the respiration and decomposition of sugar, starch and protein by the population of microorganisms. The increment in temperature is a good indicator that there is microbial activity in the compost pile, as a higher temperature denotes greater microbial activity [17]. The temperature pattern showed that there is a rapid progress from the initial mesophilic phase to the thermophilic phase for both these treatments, which points to a high proportion of readily degradable substances, e.g. vegetables and fruits (contained in a green waste material). According to Haug [18], the composting temperature has to be above 55°C for three consecutive days to kill the pathogen. Although both the compost pile treatments met the requirement, pile C_1_ has the longest period of time above 55°C, that is, for 6 consecutive days, while C_2_ only remained for three consecutive days. Even though no microbial test was done on the sample, it can be assumed that the compost treatment with EM (C_1_) has high microbial activity compared to C_2_ based on the high temperature achieved.

The declining TOC value, which was 49% for C_1_ and 36% for C_2_, is similar to that reported by Benito *et al.*[19]. According to Tumuhairwe *et al.*[16], large TOC losses suggest pronounced microbial activity in the former. The *t*-test indicated that the EM application had a significant difference (p < 0.05) on the compost, which means that treatment C_1_ lost more carbon (C) than C_2_ during the composting process. Rice straw is a material that consists of high C content. Diaz *et al.*[17] reported that during composting, C is a source of energy for microorganisms to build up cells. Almost all of the C is absorbed by the microorganisms and transformed to CO_2_ during the metabolism process of the cells. The left over C will be changed into membrane and protoplasm form. Throughout the composting process this organic matter is decomposed by microorganisms through which the organic carbon will be oxidized in aerobic condition to CO_2_ gas to the atmosphere and thus lower the C/N ratio.

The increase in the total nitrogen (N) contents at the end of the composting process differs from the values obtained by Tognetti *et al.*[20] who found that total N decreases overtime. According to Viel *et al.*[21] the increase in total N may be due to the dry mass net loss as the loss of organic C as CO_2_ during composting. In addition, the N values might also increase due to the nitrogen-fixing bacteria activity that commonly occurs at the end of composting [22]. Although a decrease of N can occur due to leaching of NO^−^_3_-N and ammonia volatilization, in this experiment, both piles were covered with plastic to retain the moisture and avoid moisture from outside, which could lead to the result obtained. Moreover, in high technology composting, where they can control the leaching problem, the resultant compost achieves an increment in total N [23,24]. The dependent pair *t*-test done on the treatments showed that the application of EM also had a significant (P < 0.05) positive effect on the net mineralization of N. The increase in the N value at the end of the composting period might occur due to the usage of N by microorganisms to build up cells, thus reducing the N, and some of the organisms will eventually die, which will be recycled as N and thus contribute to the increase [25]. The increased amount of N at the end of composting is due to this stored source of N.

The phosphorus (P) content decreases in both treatments where the P value in the C_1_ compost dropped by 4.3% while C_2_ dropped by 19%. These results are in accordance with other previous reports [26-28]. According to Tumuhairwe *et al.*[16], the loss of P during the composting process is possibly due to the leaching of P in the soluble organic solute. In contrast, there are other reports that obtain an increase in P value [29-32]. From these studies, there is a significant (P < 0.05) difference between the two treatments. Although both piles decrease in P value, the final output of C_1_ treatment has the potential to produce compost with a P value higher than the C_2_ treatment.

K is known as the element that is easily leached out [25]. However, in this study the K value increased to 55% in the C_1_ compost compared to 17% in C_2_, which shows that the compost with EM are significantly (P < 0.05) higher than without EM. The use of rice straw as a medium that can absorb moisture and maintain its structural integrity and porosity, might avoid the loss of K in compost [31]. Both treatments (C_1_ = 1.7; C_2_ = 1.4) pass the recommended value of K in compost, which is 1%. K plays an important role in plant growth where its function is to increase the elongation of the root, control ion balance, improve protein synthesis, encourage enzyme reaction, and improve the photosynthesis process and food development [33].

The accumulation of heavy metal (Zn and Fe) increases during composting, except for Cu whereas the values decrease proportional to the time. The increase of Zn and Fe are in line with previous research done by Paré *et al.*[34]. According to both reports, during composting the accumulation of nutrient and heavy metals significantly increased, which indicates the maturity of the product. Pare *et al.*[34] also concluded that the stability of biosolid compost can be correlated with the accumulation of heavy metals and nutrients, and, thus extractability and exchangeability of heavy metals.

In both treatments, the final Zn value increased but varied between the initial days until the end. A small increment was achieved by these two treatments, which are only 2% for C_1_ and 6% for C_2_. However, both composts are in the safe limit set by the European Standard [35] for heavy metals in compost, that is, in the range of 210–4000 mg kg^−1^. The decrease in Cu values in this study is similar to the result obtained by Md Sabiani [33]. Both treatments show that the decline of Cu is proportional to the days of composting, however, for the C_2_ treatment, there is an increase for day 45 before it decreases again, which differs from pile C_1_where the Cu increases a bit at the end of the composting period. The Fe values increased in both treatments, and it was found that the Fe contained in C_1_ was much higher with a range of 560–2624 mg kg^−1^ during the composting process. This could occur due to the longer exposure to high temperature above 55°C for C_1_ compared to C_2_. The high temperature increases the loss in moisture content and C in C_1_ whereas in C_2_, the Fe accumulation is low with the range of 650–2379 mg kg^−1^. The low temperature achieved during the composting process compared to C_1_ contributed to the low accumulation of Fe. This happened due to the final resultant compost still being high in moisture content, which encourages the leaching process to occur, and, thus, reduces the Fe value. This theory correlates with the finding of Md Sabini [33] in which compost that loses its moisture content and C accumulate more Fe. The Fe also increases when there is a reduction of substrate pH in the early composting period, which increases the solubility of metals, including metallic microelements [36]. The *t*-test conducted on the heavy metals showed that there is no significant difference between the treatment with EM and without EM on the value of heavy metals except for Fe. The compost applied with EM shows a significant (P < 0.05) difference in Fe accumulation.

Compost usually contains heavy metals based on their initial raw material. Generally, these heavy metals (micronutrients) are required by the plant for perfect growth. The heavy metals present in the compost, such as Zn, Cu and Fe, are absorbed by plants during the fertilizing process. In a small quantity, these trace elements are necessary for plant growth but in large quantities they can cause phytotoxicity [37]. Kabata-Pendias and Pendias [38] also described in their research that Zn, Cu, Mn and Fe are useful as trace elements for crop growth. However, constant and intense application of organic compost containing heavy metals will lead to accumulation in the soil and an increase in toxic levels.

## Conclusion

In general, all the composting parameters show that both treatments have a similar pattern. All the parameters measured indicate that a decomposition process occurs in both treatments. The parameters for the temperature and pH show that the decomposition of organic matter occurs during the 90-day period. The decrease in TOC values and C/N ratio also shows that an organic compound is being consumed by microorganisms. The *t*-test conducted shows that there is a significant difference between the compost treated with EM and the compost without EM. The application of EM in compost increased the macro and micronutrient content. The following parameters support this conclusion: compost applied with EM has more N, P and K content (P < 0.05) compared to compost without EM. While the Fe in compost with EM is much higher (P < 0.05) than compost without EM. However, for Zn and Cu, there is no significant difference between treatments. All of the micronutrient (heavy metals) values are below the standard limit of heavy metals in compost.

## Competing interests

The authors declare that they have no competing interests.

## Authors’ contributions

MLCJ has designed and performed experiments, analyzed data and wrote the manuscript. LAM has guide in the experiments design, suggestion of analysis and manuscript preparation. PAL has guided in the laboratory analysis, interpretation techniques as well as manuscript preparation. All authors read and approved the final manuscript.
